# Analysis of molecular mechanism for acceleration of polyembryony using gene functional annotation pipeline in *Copidosoma floridanum*

**DOI:** 10.1186/s12864-020-6559-3

**Published:** 2020-02-11

**Authors:** Takuma Sakamoto, Maaya Nishiko, Hidemasa Bono, Takeru Nakazato, Jin Yoshimura, Hiroko Tabunoki, Kikuo Iwabuchi

**Affiliations:** 1 Department of United Graduate School of Agricultural Science, Tokyo University of Agriculture and Technology, Tokyo, Japan; 2 Department of Science of Biological Production, Graduate School of Agriculture, Tokyo University of Agriculture and Technology, Tokyo, Japan; 3 Database Center for Life Science (DBCLS), Joint Support-Center for Data Science Research, Research Organization of Information and Systems (ROIS), Mishima, Shizuoka Japan; 4 Department of Mathematical Systems Engineering Shizuoka University, Hamamatsu, Japan; 5 Department of Environmental and Forest Biology, State University of New York College of Environmental Science and Forestry, Syracuse, New York USA; 6 Marine Biosystems Research Center, Chiba University, Kamogawa, Chiba, Japan

**Keywords:** Polyembryony, Xanthine dehydrogenase/oxidase, Endoparasitoid, Polyembryogenesis, *Copidosoma floridanum*

## Abstract

**Background:**

Polyembryony is defined as the formation of several embryos from a single egg. This phenomenon can occur in humans, armadillo, and some endoparasitoid insects. However, the mechanism underlying polyembryogenesis in animals remains to be elucidated. The polyembryonic parasitoid wasp *Copidosoma floridanum* oviposits its egg into an egg of the host insect; eventually, over 2000 individuals will arise from one egg. Previously, we reported that polyembryogenesis is enhanced when the juvenile hormone (JH) added to the culture medium in the embryo culture. Hence, in the present study, we performed RNA sequencing (RNA-Seq) analysis to investigate the molecular mechanisms controlling polyembryogenesis of *C. floridanum*. Functional annotation of genes is not fully available for *C.floridanum*; however, whole genome assembly has been archived. Hence, we constructed a pipeline for gene functional annotation in *C. floridanum* and performed molecular network analysis. We analyzed differentially expressed genes between control and JH-treated molura after 48 h of culture, then used the tblastx program to assign whole *C. floridanum* transcripts to human gene.

**Results:**

We obtained 11,117 transcripts in the JH treatment group and identified 217 differentially expressed genes compared with the control group. As a result, 76% of *C. floridanum* transcripts were assigned to human genes. Gene enrichment analysis revealed genes associated with platelet degranulation, fatty acid biosynthesis, cell morphogenesis in the differentiation and integrin signaling pathways were fluctuated following JH treatment. Furthermore, Cytoscape analysis revealed a molecular interaction that was possibly associated with polyembryogenesis .

**Conclusions:**

We have constructed a pipeline for gene functional annotation of *C. floridanum*, and identified transcripts with high similarity to human genes during early embryo developmental. Additionally, this study reveals new molecular interactions associated with polyembryogenesis; these interactions could indicate the molecular mechanisms underlying polyembryony. Our results highlight the potential utility of molecular interaction analysis in human twins.

## Background

The development of a single-cell egg into a multicellular organism begins with cell cleavage; however, polyembryogenesis—in which many embryos are produced from one egg—occurs in some species. Although identical twins are representative examples of human polyembryogenesis, the incidence of this situation is as low as 0.3% [1]. Armadillos (*Dasypus*) are the only mammals to exhibit obligatory polyembryony, developing from one egg to four individuals through embryonic shield separation [2, 3]. Owing to the ethical limitations of experiments with animal, the phenomenon of polyembryogenesis remains poorly studied.

Furthermore, the occurrence of polyembryogenesis has been reported for insects such as the endoparasitic wasps Braconidae, Dryinidae, Platygasteridae, and Encyrtidae, and research on developmental patterns has progressed rapidly in recent years [4]. The identification of molecules associated with polyembryogenesis could further our understanding of the mechanisms underlying the regulation of this process in animals.

**Fig. 1:**
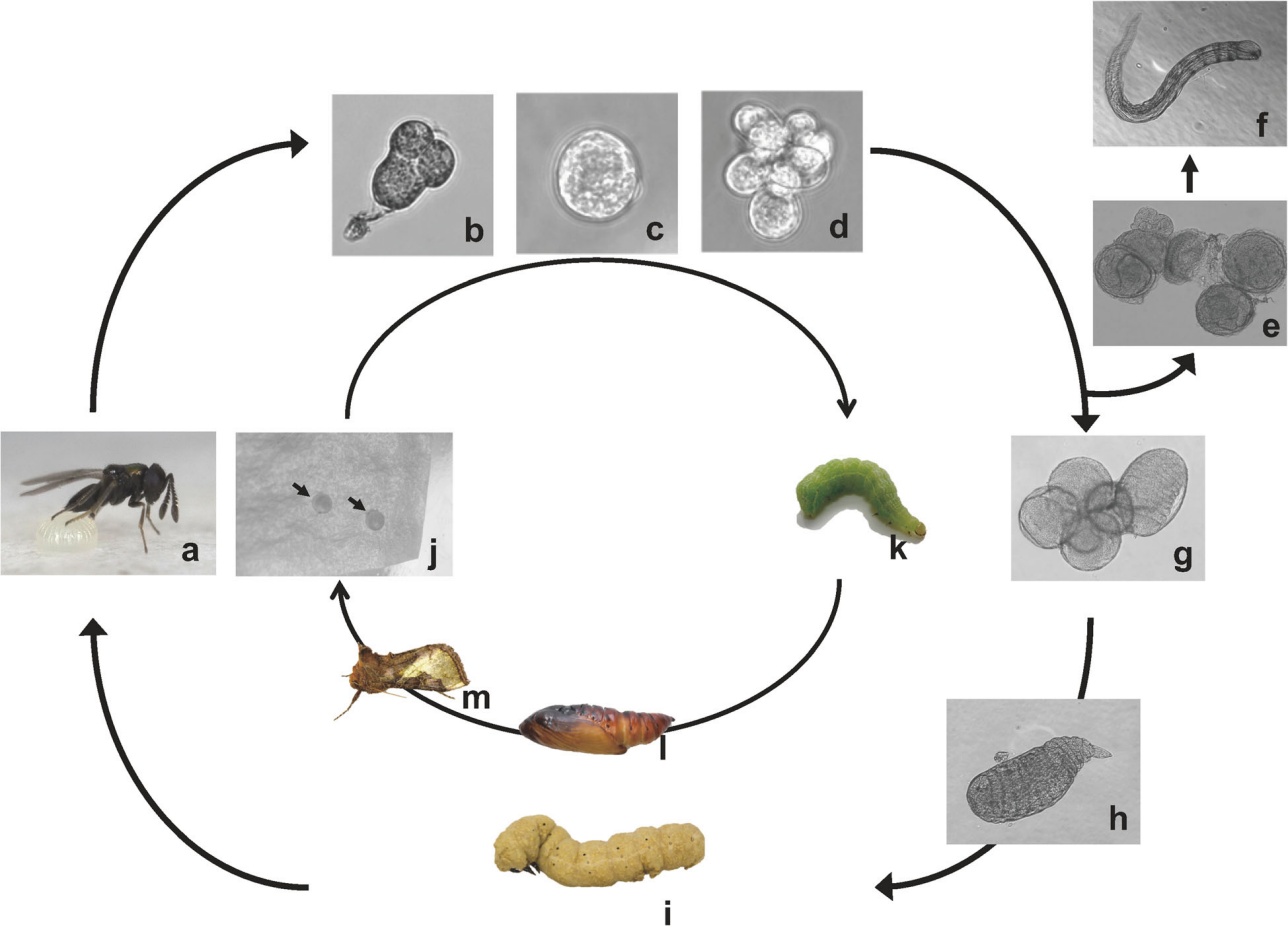
The life cycle of *Copidosoma floridanum*. *C. floridanum* oviposited its egg into the host egg (**a**). Then, the *C. floridanum* egg starts developing from the two-cell stage to morula and invaginates into host the embryo (**b** and **c**). The morula embryo is clonally divided, and polyembryos are formed around 60 h after parasitism after invading the host embryo (**d**). A part of polyembryos starts segmentation and, then, develops into soldier larvae via soldier embryo (**e** and **f**). Each embryo achieves morphogenesis when the host insect develops at the end of the fifth instar larvae (**g**) and, then, reproductive larva appears (**h**). Finally, reproductive larvae emerge when the host insect achieves on the second day of sixth instar larvae, and the adult emerges from the mummy (**i**). j-m: *T. intermixta* life cycle. (**j**) *T. intermixta* egg indicates black arrows. (**k**) final (sixth) instar larva. (**l**) pupa, (**m**) adult

The endoparasitic wasp *Copidosoma floridanum* (Hymenoptera: Encyrtidae) is an egg-larval parasitoid of the plusiine moth *Thysanoplusia intermixta*. The egg developmental stage of *T. intermixta* lasts 4 days, the larval developmental stage around 20 days, and pupal developmental lasts around 8 days under experimental conditions [5] (Fig. 1). In standard experimental conditions, *C. floridanum* parasitizes the 2-day old egg of *T. intermixta*, ultimately producing nearly 2000 cloned embryos from either a fertilized egg (which develops into females) or an unfertilized egg (which develops into males) (Fig. 1). Although almost all insects exhibit egg segmentation due to superficial cleavage, *C. floridanum* egg segments undergo holoblastic cleavage. In *C. floridanum*, cell cleavage begins when the egg is laid into a host egg (Fig. 1a). The *C. floridanum* egg then starts to develop from the two-cell stage to a morula after the *C. floridanum* embryo invades the host embryo (Fig. 1b). The egg of *C. floridanum* comprises an embryonic cell and an anterior cell from a polar body [6]. The anterior cell develops into an extraembryonic syncytium, which forms the outer part of the morula. The extraembryonic syncytium from the anterior cell wraps around the blastomeres after the chorion is removed and morula form (Fig. 1c). The motility of early morulae enable them to invade the host embryo and then develop into polyembryos when morulae movement ceases after entry into the host embryo [7] (Fig. 1d). A subset of polyembryos starts to develop into soldier larvae from embryos without primary germ cells (Fig. 1e and f). Each embryo undergoes morphogenesis when the host insect reaches the end of the final instar of larval development (Fig. 1g). Finally, reproductive larvae emerge when the host insect reaches the second day of the final instar of larval development (Fig. 1h), and adults finally emerge (Fig. 1i) [8, 9]. Although the process by which *C. floridanum* polyembryony progresses in host embryos is known, the molecular mechanisms remain obscure.

Previously, we reported polyembryogenesis to be accelerated by exposure to juvenile hormone (JH) or the JH analog methoprene, under culture conditions [10]. Although the molecular function of JH in the embryogenesis of *C. floridanum* remains unclear, we anticipated that either JH or methoprene would promote the progress of polyembryogenesis from the two-cell to polymorula stage. Functional annotation of genes is not fully available for *C. floridanum*; however, whole-genome sequence data is available. Thus, we were unable to analyze the molecular networks in *C. floridanum* as has been done for *Drosophila melanogaster*, a model insect. Accordingly, we constructed a pipeline for gene functional annotation of *C. floridanum* and performed molecular network analysis focusing on gene expression following polyembryony development in response to JH treatment in the two-cell developmental stage. We also performed RNA sequencing (RNA-Seq) analysis of *C. floridanum* as a model animal for polyembryogenesis to elucidate the molecular mechanisms underlying this process.

## Results

### Juvenile hormone accelerates polyembryogenesis

**Fig. 2:**
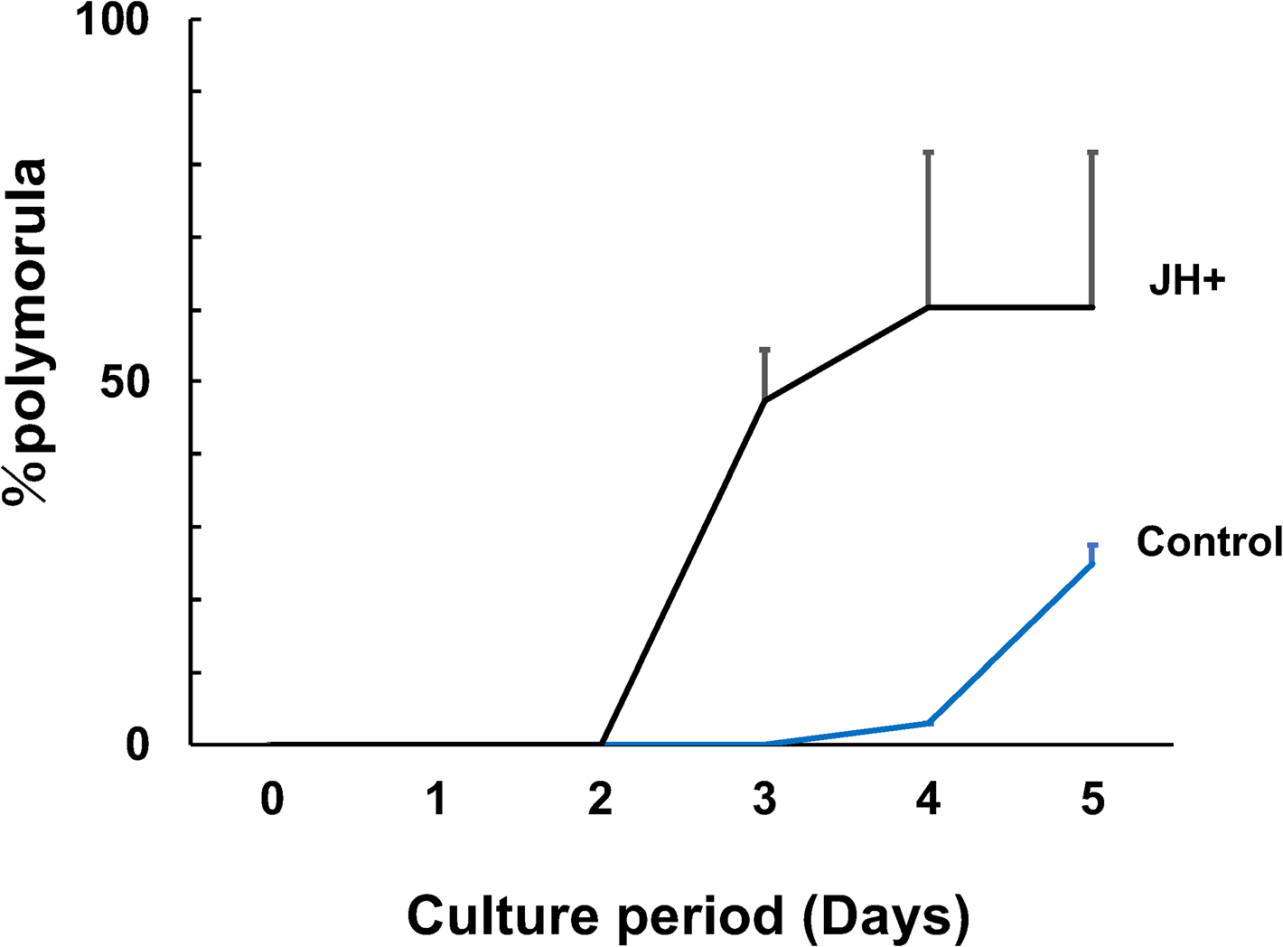
Polymorula was accelerated by the juvenile hormone (JH) treatment in the culture condition. The early embryo of the two-cell stage was cultured with or without JH. The number of polymorula was counted and plotted on the graph. Notes: Black line, JH-treated group; blue line, control group; vertical axis, rate of polymorula (%); horizontal axis, culture period (days). Error bars indicate standard deviation (SD)

We collected the two-cell-stage embryos and cultured them for 5 days; then, we counted the cultured embryo and evaluated whether these embryos were polymolurae or not (Fig. 2). We found the JH-treated group to exhibit an increased rate of polymolurae compared with the control group. Additionally, polymolurae were identified in the JH-treated group from 2 days after the start of culturing, whereas these were only identified in the control group after 4 days after culture. Hence, the development of polymolurae was accelerated by JH treatment (Fig. 2; Table 1).

**Table 1:** Impact of polyembryogenesis by the juvenile hormone treatment to the early embryo of *Copidosoma floridanum*

Treatment groups	Day of polymorula	Rate of polymorula (%)
Control	4.88 ± 0.13	25.0
JH	3.16 ± 0.23	60.3

Control group: experimental 1, *n* = 10; experimental 2, *n* = 25; experimental 3, *n* = 21. Juvenile-hormone-treated group (experimental 1, *n* = 10; experimental 2, *n* = 24; experimental 3, *n* = 23). Polymorula was observed in experimental 1 to 3 groups and the day of polymorula is shown as mean ± standard deviation. *Abbreviations*: *JH* juvenile hormone

### Identification of differentially expressed genes and assignment of human homolog

**Fig. 3:**
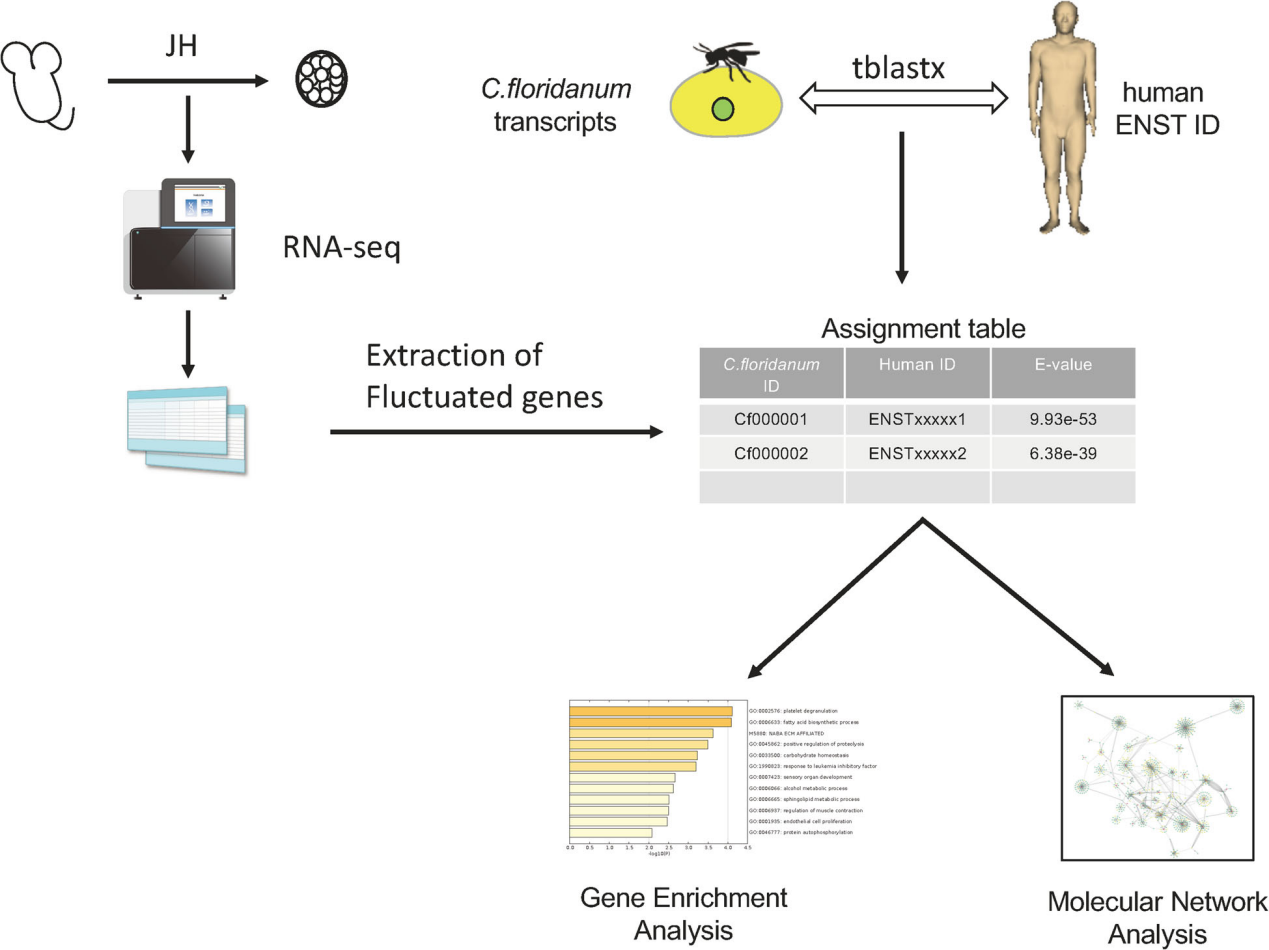
The strategy of annotation of *Copidosoma floridanum* transcript to human genes. We performed tblastx between *C. floridanum* transcripts and human transcripts on public databases to extract fluctuated genes in the juvenile hormone (JH) treatment using the RNA-Seq analysis. Then, we converted transcripts IDs from the *C. floridanum* RNA-Seq to human Ensembl IDs and constructed assignment table. Finally, we performed the gene enrichment analysis and molecular network analysis using the public database and determined molecular interaction in the polyembryogenesis. The experimental tools, human and machines drawings (https://togotv.dbcls.jp/ja/pics.html) are licensed under Creative Commons Attribution 4.0 International License (CC BY 4.0) (http://creativecommons.org/licenses/by/4.0/deed.en)

We constructed a pipeline for functional gene annotation of *C. floridanum* (Fig. 3). In the present study, we identified 13,160 human homologs of 17,308 total predicted protein dataset in *C. floridanum* using the current gene functional annotation pipeline. As a result, *C. floridanum* exhibits 76% gene similarity with humans. Following the results of polymurae analysis, we extracted total RNA for RNA-Seq analysis after 48 h of cell culture. We performed RNA-Seq analysis on three control samples (SRA accession numbers: DRR138914, DRR138915, and DRR138916) and two JH-treated samples (DRR138917 and DRR138918). The RNA-Seq data were mapped with HISAT2 and StringTie. We obtained 11,117 transcripts from these RNA-Seq data and found that 10,908 of these were commonly expressed in the control and JH-treated groups.

**Fig. 4:**
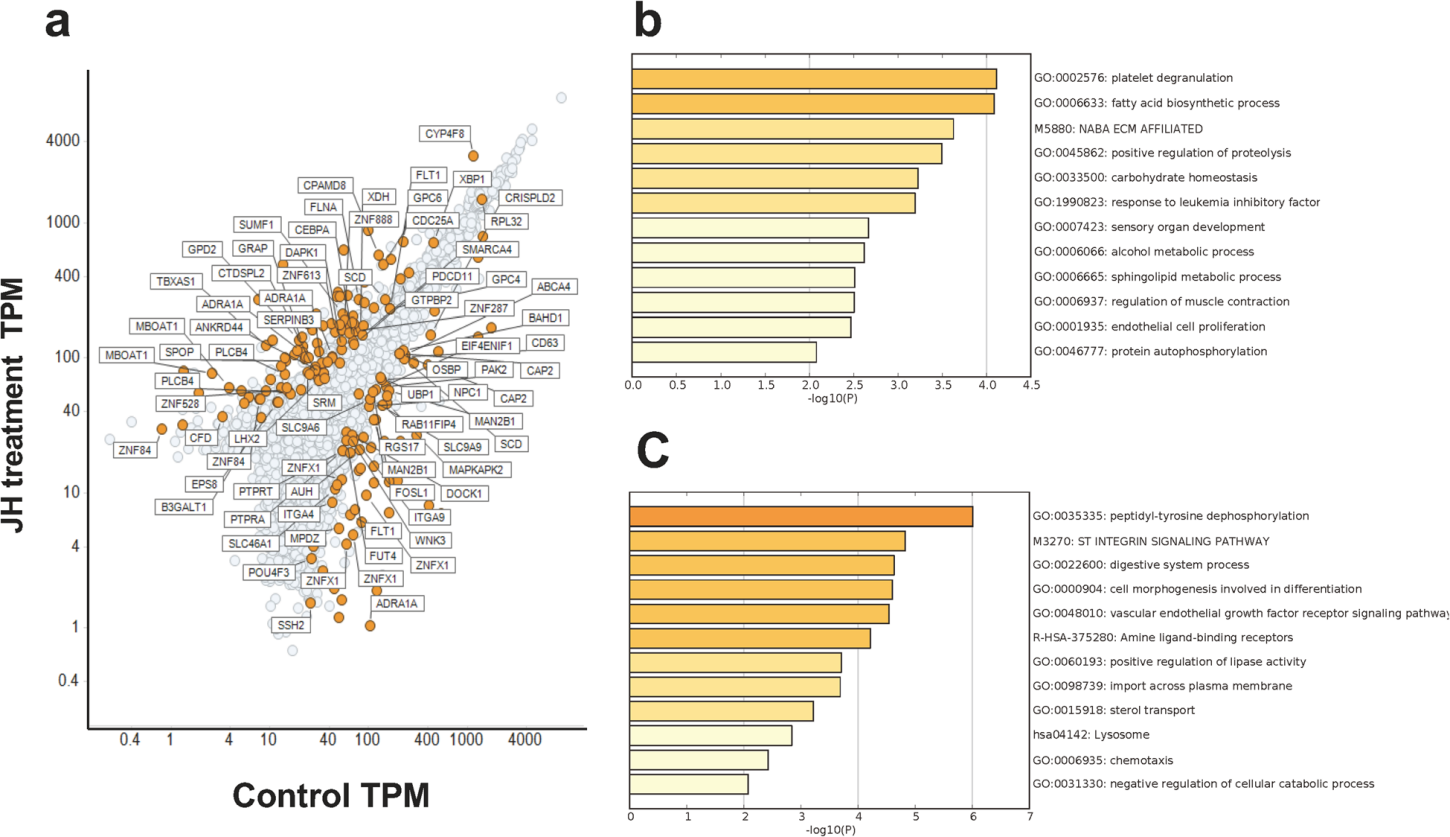
The extraction of fluctuated transcripts in the juvenile hormone (JH) treatment and gene enrichment analysis. Fluctuated transcripts were extracted and plotted on the graph. Orange dots, transcripts that fluctuated more than two times compared with the control in the JH treatment (**a**). The gene enrichment analysis of fluctuated transcripts in the morula using Metascape. A heatmap of enriched terms across the input transcripts lists; different colored bars, *P* values. (**b**) JH upregulated genes; (**c**) JH downregulated genes

We identified 217 differentially expressed genes (DEGs; false discovery rate [FDR] < 0.05; Fig. 4a). While the expression of 123 of these was increased, 94 were downregulated in the JH-treated group (Fig. 4a, orange-colored dots indicate differentially expressed transcripts).

Next, we assessed the number of *C. floridanum* genes by comparing our transcript dataset with the *C. floridanum* RefSeq datasets. Of the 11,117 transcripts in our *C. floridanum* RNA-Seq datasets, 9417 were present in the *C. floridanum* RefSeq datasets (84.7% coverage). We identified 6098 human homologs out of the 11,117 total transcripts of *C. floridanum* using tblastx with a cutoff *E*-value of 1e–10. Of the 217 DEGs that we identified, 88 corresponded to human genes (Additional file 1: Table S1).

### Gene enrichment analysis of differentially expressed genes

For the gene enrichment analysis using Metascape, we needed to assign human gene IDs to *C. floridanum* genes IDs. Thus, we chose *C.floridanum* 88 genes, which have sequence similarity to human genes. Of 88 genes that were chosen to compare expression between the two groups, the expression of 42 genes was increased in the JH-treated group, whereas 46 were downregulated following JH treatment. We imported the list of DEGs and their expression levels into Metascape and converted to their human homologs for gene enrichment analysis. Metascape generated 12 genetic function groups for Gene Ontology (GO), indicating that upregulated genes in the JH-treated group were assigned to platelet degranulation (GO:0002576) and fatty acid biosynthetic process (GO:0006633) (Fig. 4b). Furthermore, Metascape indicated that downregulated genes in the JH-treated group were assigned to cell morphogenesis was involved in differentiation (GO: 0000904) and ST Integrin Signaling Pathway (M3270) (Fig. 4c).

### Screening for related molecules using the molecular network analysis by Cytoscape

**Fig. 5:**
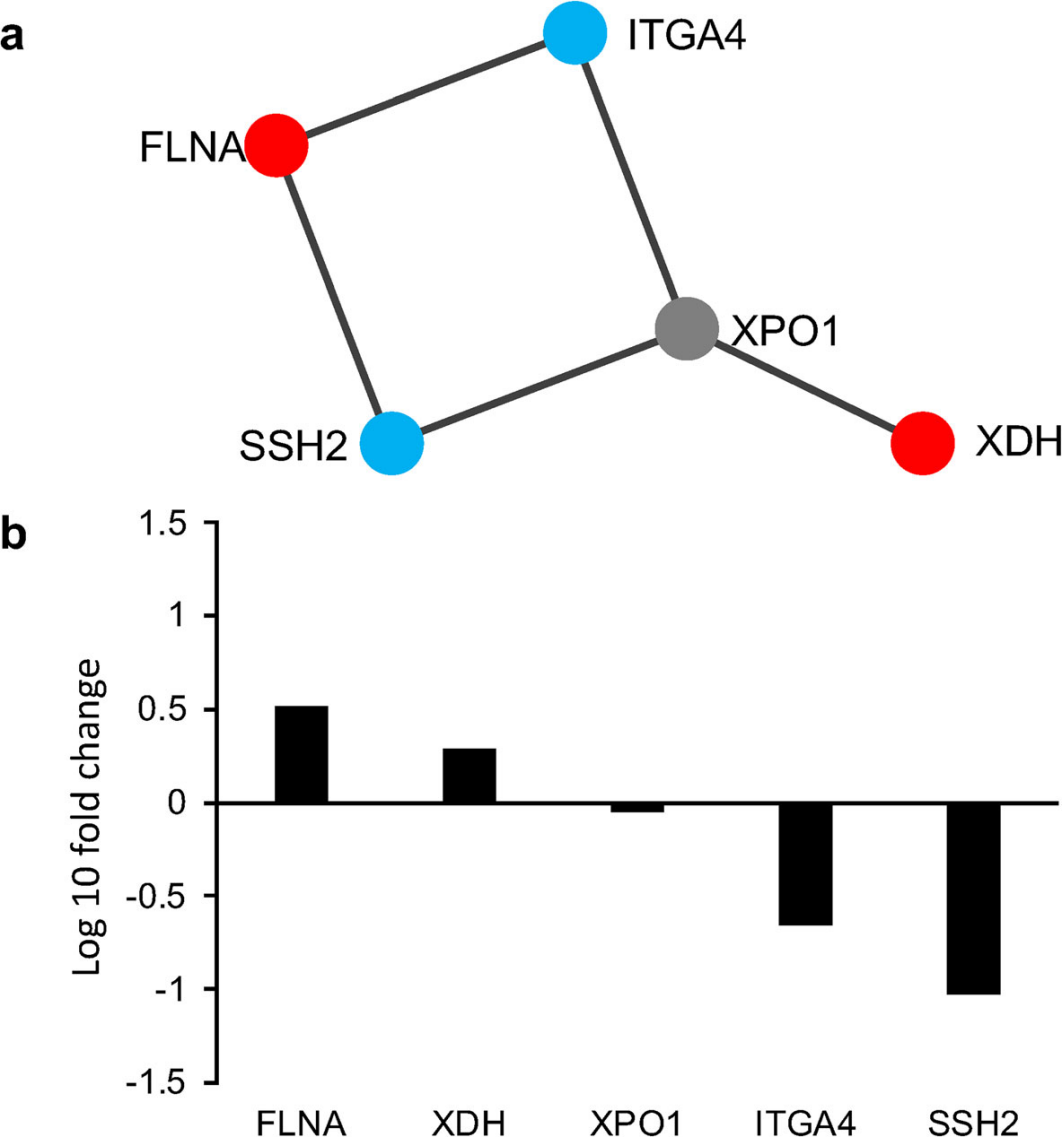
Screening of the molecular interaction using Cytoscape. The molecular interaction of (**a**) Cytoscape automatically generates the corresponding molecules as a node on a molecular. Interaction in polyembryogenesis-related genes. A red colored node shows up-regulated genes, blue colored genes shows down-regulated genes, and a gray colored gene is not fluctuation gene. (**b**) Polyembryogenesis-related genes in juvenile hormone (JH) treatment of molura. The *y*-axis indicates the ratio of the average Transcripts Per Kilobase Million (TPM) values for molura between the control and JH treatment groups

We focused on the GO terms platelet degranulation, fatty acid biosynthetic process, and integrin signaling pathway to identify the molecular network that regulates polyembryogenesis. Using the public protein interaction database and Cytoscape, we further explored correlations among the genes involved in these processes. Hence, we identified molecular interactions including filamin-A (*FLNA*), xanthine dehydrogenase/oxidase (*XDH*), exportin-1 (*XPO1*), protein phosphatase slingshot homolog 2 (*SSH2*), and integrin alpha-4 (*ITGA4*) as genes that fluctuated in the JH-treated group (Fig. 5a). The Transcripts Per Kilobase Million (TPM) values of these molecules were plotted (Fig. 5b). We found that the mRNA expression of *FLNA* and *XDH* increased, while that of *SSH2* and *ITGA4* decreased following JH treatment (Fig. 5b). However, the mRNA expression of *XPO1* was not by JH treatment (Fig. 5b).

## Discussion

We investigated gene expression during polyembryogenesis of embryos of *C. floridanum* treated with JH in the two-cell stage. Additionally, we constructed a pipeline for functional gene annotation of *C. floridanum* and performed molecular network analysis.

Our previous study revealed that JH and methoprene accelerate polyembryogenesis; a phenomenon that was also observed when JH I or JH II were added to the culture medium [10]. Methoprene had the strongest effect on promotion of polyembryogenesis, while farnesol, farnesyl acetate, and methyl caproate did not promote polyembryogenesis [10]. Moreover, polyembryogenesis was not promoted by ecdysone (Additional file 2: Figure S1). Hence, only JH or methoprene promote polyembryogenesis in *C. floridanum*.

The insect hormone JH is unique in its structure; it has α-, and β-unsaturated methyl ester groups and epoxy groups at both ends of the terpenoid backbone [11]. The importance of JH in processes such as regulation of molting, pheromone biosynthesis, maturation of gonads, egg development, homeostasis, maintenance of population, and body color change has been reported [12]. Thus, JH is a critical element of insect physiology.

Krüppel homolog 1 (*Kr-h1*) is a JH-responsive gene [13]. *Kr-h1* was not affected by JH in this study (Additional file 3: Figure S2). Retinoid X receptor (RXR), a type of nuclear receptor that binds to 9-cis retinoic acid [14], may be able to bind to several chemicals that share the structure of retinoic acid [15]. Reportedly, the JH analog methoprene and methoprene acid can bind to RXR [15], which also functions as a JH receptor in *D. melanogaster* [16]. In this study, the expression of *C. floridanum* RXR-alpha-B tended to be increased following JH treatment (Additional file 3: Figure S2). Retinoic acid is involved in embryonic development and cell differentiation through its interaction with RXR [17]. This suggests that JH could act in a similar manner to retinoic acid to affect embryonic development and participate in the retinoic acid signaling pathway in our model of polyembryony.

Additionally, *C. floridanum* RefSeq datasets were constructed by analyzing male and female adult *C. floridanum* transcriptome. A reference genome sequence of *C. floridanum* from male has been published (https://www.ncbi.nlm.nih.gov/assembly/GCF_000648655.2/). Overall, 17,038 of the estimated proteins have been registered in the NCBI Genome database (https://www.ncbi.nlm.nih.gov/genome/12734?genome_assembly_id=358239).

Previously, we identified human homologs of *Bombyx mori* and *D. melanogaster* through systematic BLAST analysis. We found *B. mori* and *D. melanogaster* to contain 58 and 63% of human homologs, respectively [18]. In the present study, we found, *C. floridanum* exhibits 76% gene similarity with humans. The large number of human homologs that we observed in *C. floridanum* is therefore comparable with these model insects. In the present study, 88 *C. floridanum* genes that showed differential expression when comparing JH-treated and control groups corresponded to human genes, and we input these genes to gene enrichment and molecular network analyses.

Gene enrichment analysis revealed the expression of lipid metabolism-related (GO: 0006633) and platelet degranulation (GO: 0002576) related genes were upregulated in the JH-treated group. The characteristics of genes containing these GO terms have been shown to be involved with vesicle-associated V-soluble N-ethylmaleimide-sensitive factor attachment protein receptor (SNARE) in cellular membrane adhesion [19]. Additionally, genes encoding synaptosomal-associated protein (*SNAP*)25 and 29, (*SNAPC*) 3 and 4, SNARE-associated protein Snapin (*SNAPIN*), and syntaxin-binding protein (*STXBP*) were shown to be commonly expressed in both groups of the present study. Reportedly, these genes are involved in the membrane fusion of neurosecretory cells [20]. Notably, STXBP interacts with these proteins to inactivate membrane fusion [20]. Thus the decreased mRNA expression of *STXBP* that we observed following JH treatment may lead to activation of cellular membrane fusion. As the molura promotes fusion with the extraembryonic syncytium, it is possible that JH treatment could accelerate morula to polyembrogenesis.

The genes *SNAP25* and *SNAP29*, *SNAPC3* and *SNAPC4*, *SNAPIN,* and *STXBP,* which we observed to be expressed in *C. floridanum* morula embryos, also play a role for mediates membrane fusion during exocytosis in the neurosecretory cells of humans [21]. Therefore, *C. floridanum* might employ similar molecular mechanisms for cellular membrane adhesion as human neurosecretory cells.

The morula comprises the outer extraembryonic syncytium and the inner embryonic cell; the extraembryonic syncytium separates by dividing the embryonic cell [22]. Embryonic cells adhere to the extraembryonic syncytium via integrin in the morula. When integrin expression is decreased following exposure to JH, this adhesion may be loose, causing the extraembryonic syncytium to invaginate into the cell gap of embryonic cells. These cells might then divide, resulting in polyembryony. The actin filament crosslinking protein, FLNA, which is present in non-muscle cells [23] might be involved in the division of embryos interacting with *SSH2* and *ITGA4*. Reportedly, *SSH2* mediates actin dynamics [24], while ITGA4 belongs to the integrin family and plays a role in cell adhesion [25]. These molecular interactions might contribute to the polyembryony.

Xanthine dehydrogenase/oxidase is the rate-limiting enzyme of purine metabolism, and a key component in uric acid synthesis. During egg development of *B. mori* the amount of uric acid gradually declines until blastokinesis, after which it increases until egg pigmentation [26]. Remarkably, XHD knockdown has been shown to enhance cell mobility and invasion of HepG2 cells, although no effect on cell proliferation was observed [27]. Furthermore, XDH converts retinoic acid to 9-cis retinal [28], and activity of the enzyme is essential in order for JH to induce bristle formation and cuticle production on the abdominal epidermis during pupal and adult development [29].

**Fig. 6:**
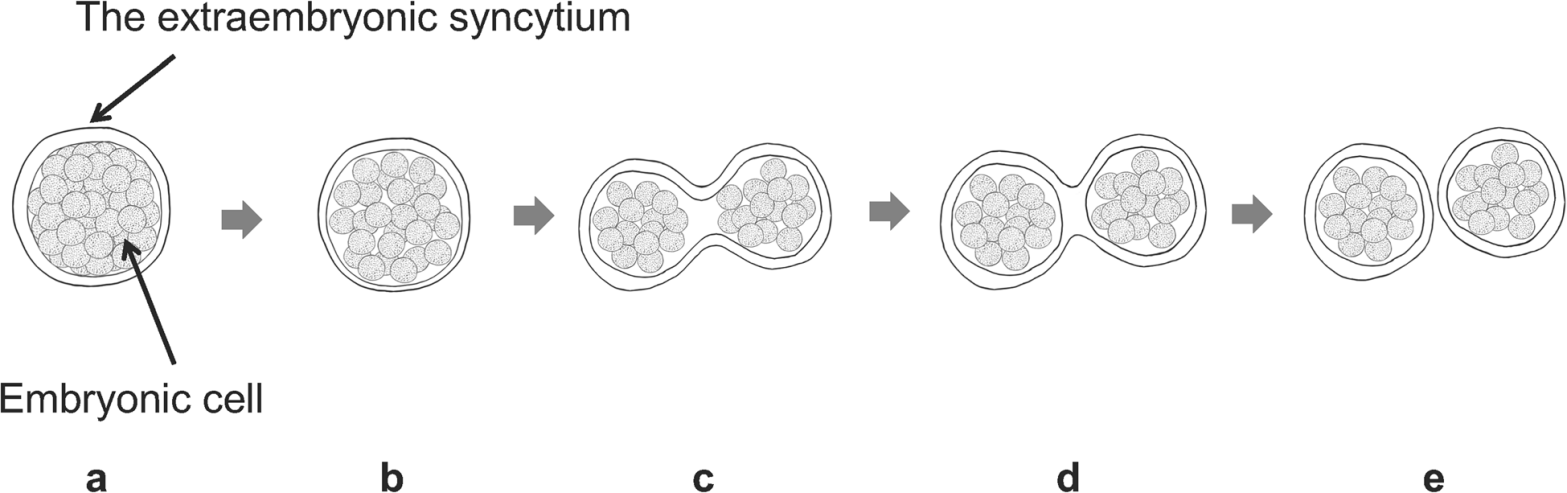
The model for the molecular mechanism of polyembryogenesis in *Copidosoma floridanum.* **a** The embryonic cell and the extraembryonic syncytium are attached in the molura. **b** The juvenile hormone (JH) loosens the cell attachment between the embryonic cells and the extraembryonic syncytium. **c** The extraembryonic syncytium invaginate into the molura. **d** After the inside of the extraembryonic syncytium has fused, **e** each cell is separated and polymorula are formed

The chemical structures of JH III and retinoic acid are similar, and it is possible that JH III binds to XDH. Then, XDH-bound JH could enter the nucleus via *XPO1*, and released JH might bind to RXR to control subsequent transcription; this molecular correlation could be a novel mechanism triggered by JH. Accordingly, the following model could be proposed for the progression of forming molura to polymolura: To start, embryos are wrapped with extraembryonic syncytium (Fig. 6a). During the formation of polymolurae, the degradation of actin is avoided by reducing *SSH2* expression followed by suppression of *ITGA4* expression. As the adhesion of embryonic cells is loose (Fig. 6b), the syncytial membrane facilitates fractionation (Fig. 6c and d) and promotes polyembryony (Fig. 6e). Hence, XDH plays a key role in embryogenesis via JH, as well as in uric acid synthesis. Overall, the present study reveals novel molecular interactions involved in polyembryogenesis and demonstrates the connections that are required for the progression of cell separation in polyembryogenesis.

## Conclusions

In this study, we constructed a pipeline for gene functional annotation of *C. floridanum.* We identified *C. floridanum* transcripts with high similarity with several human genes during early embryogenesis, and found that *C. floridanum* has many more human homologs than *D. melanogaster*. Additionally, we identified new molecular interactions associated with polyembryogenesis, which may enable the elucidatation of the molecular mechanisms underlying polyembryony. Our results highlight the potential utility of molecular interaction analysis for the investigation of polyembryogenesis of humans. In future studies, we intend to investigate the function of these molecules using RNAi in *C. floridanum*.

## Methods

### Insect

We obtained *C. floridanum* from parasitized *T. intermixta* larvae from burdock fields in Tokyo, Chiba, and Ibaraki Prefectures. Larvae were maintained with an artificial diet [30] at 25 °C with a 16- h light/8-h dark cycle. *T. intermixta* adults were fed a 10% sugar solution absorbed with cotton. *C. floridanum* adults were fed a 50% honey solution absorbed with cotton. We used *T. intermixta* eggs within 17 h post-oviposition for parasitism by *C. floridanum*. The parasitized hosts were kept in the same conditions as non-parasitized hosts.

### Embryo culture

We dissected the two-cell stage of the embryo from *T. intermixta* eggs within 2-h post-oviposition and, then, cultured it with the modified MGM medium [31]. We dissolved JH III (J2000; Sigma-Aldrich Co. Ltd., Munich, Germany) in ethanol to prepare a stock solution (10 mg/mL); then, 1 μL of this stock solution was added to the modified MGM medium as the final concentration was 1 μg/mL. In the control group, an equivalent volume of ethanol alone was added to the culture medium. We used two-cell stage embryos produced from *T. intermixta* eggs within 2 h of oviposition for the control group (experimental 1, *n* = 10; experimental 2, *n* = 25; experimental 3, *n* = 21) and the JH group (experimental 1, *n* = 10; experimental 2, *n* = 24, experimental 3, *n* = 23).

### RNA-Seq analysis

We isolated total RNA from morulae cultured for 2 days in control (experimental 1, *n* = 24; experimental 2, *n* = 16; experimental 3, *n* = 11), and JH conditions (experimental 1, *n* = 17; experimental 2, *n* = 28; experimental 3, *n* = 23) using a combination of TRIzol® LS Reagent and the PureLink® RNA Extraction Kit (Thermo Fisher Scientific Inc., Valencia, CA) per the manufacturer’s instructions. Then, we used an Agilent TapeStation 2200 (Agilent Technologies, Santa Clara, CA) to assess the RNA quality. Additionally, single-end sequencing cDNA libraries were constructed with 100 ng of total RNA from these samples (control group: *n* = 3; JH group: *n* = 3) with a TruSeq® Stranded mRNA Sample Preparation Kit (Illumina Inc., San Diego, CA) per the manufacturer’s instructions. Next, libraries were sequenced (75 bp, single-end) on the Illumina NextSeq500 platform, and FASTQ files were assessed by Trim Galore! v0.4.5 (https://www.bioinformatics.babraham.ac.uk/projects/trim_galore/). Sequencing of the JH group of experimental 3 was not possible as the creation of a library for RNA-Seq failed. Thus, we omitted the data of this group. Finally, we analyzed gene expression using three biological replicates in the control group and two biological replicates in the JH group. Notably, the *C. floridanum* genome (GCF_000648655.2) sequence is available in the NCBI Genome database (https://www.ncbi.nlm.nih.gov/genome/annotation_euk/Copidosoma_floridanum/101/). The obtained FASTQ sequence files were aligned to the genomic reference sequence by HISAT2 v2.1.0 with default parameters [32]. Next, SAM files were converted to BAM files by Samtools v1.8 [33]. Using StringTie v1.3.4, we estimated the transcript abundance, and the count data were extracted by Subread v1.6.0 [34, 35]. All statistical analyses were performed using R software version 3.4.3 (https://www.r-project.org). To normalize the data and compare the control and JH-treated groups, we used the TCC and DEseq2 packages [36]. We generated a scatter plot using TIBCO Spotfire Desktop v7.6.0 with the “Better World” program license (TIBCO Spot re, Inc., Palo Alto, CA; http://spotfire.tibco.com/better-world-donation-program/). Furthermore, the sequence data (FASTQ files) were deposited to the DDBJ Sequence Read Archive (accession numbers DRR138914-DRR138918).

### *C. floridanum* gene functional annotation pipeline for molecular network analysis

The transcripts were extracted from the *C. floridanum* genome using gffread software (https://github.com/gpertea/gffread). To annotate *C. floridanum* gene, we identified genes homologous to those of human by conducting a systematic BLAST search (tblastx) with a cutoff E-value of significant similarity at 1e–10 (query: *C. floridanum* cDNA sequence; database: whole human cDNA sequence set from Ensembl database). Using the generated assignment table, we reconstructed conserved pathways common to *C. floridanum* and humans by projecting *C. floridanum* genes onto the human pathway.

### Gene enrichment analysis and pathway analysis

We performed the gene enrichment analysis using Metascape (http://metascape.org/); a gene list for Metascape analysis was generated from the TCC output. Then, we performed the molecular network analysis using IntAct Molecular Interaction Database (https://www.ebi.ac.uk/intact/) and Cytoscape v3.6.1 (http://www.cytoscape.org). We converted the gene IDs from the *C. floridanum* RNA-Seq data to human Ensembl IDs, using the assignment table described above and, then, input the list of genes obtained from the RNA-Seq data into Cytoscape to obtain the significant molecular interactions with corresponding *E* values.

## Supplementary information

**Additional file 1: Table S1.** *C. floridanum* genes assign to the human gene. Up-regulated genes and down-regulated genes were assigned to the human gene. These genes were used for gene set enrichment analysis.

**Additional file 2: Figure S1.** Polymorula was accelerated by the juvenile hormone (JH) treatment in the culture condition. The early embryo of the two-cell stage was cultured with or without JH, or Ecdysone. The number of polymorula was counted and plotted on the graph. Solid line, JH treatment; dashed line, treatment without JH; wide dashed line, treatment Ecdysone, vertical axis, a rate of polymorula (%); horizontal axis, the culture period (day).

**Additional file 3: Figure S2.** Expression of Krueppel homolog 1-like genes, and retinoic acid receptor RXR-alpha-B in juvenile hormone (JH) treatment of molura. The *y*-axis indicates the ratio of the average Transcripts Per Kilobase Million (TPM) values for molura between the control and JH treatment groups.

## Data Availability

The RNA-seq reads supporting the conclusions of this article are available in the Sequence Read Archive (SRA) with accession IDs: DRR138914, DRR138915, and DRR138916; control samples, DRR138917 and DRR138918;JH-treated samples.

## Abbreviations

DEG: Differentially expressed genes
GO: Gene ontology
JH: Juvenile hormone
RQ: Relative quantification
RXR: Retinoid X receptor
SNARE: Sensitive factor attachment protein receptor
TPM: Transcripts per kilobase million

## References

[1] Tong S, Caddy D, Short RV (1997). Use of dizygotic to monozygotic twinning ratio as a measure of fertility. Lancet.

[2] Enders AC (2002). Implantation in the nine-banded armadillo: how does a single blastocyst form four embryos?. Placenta.

[3] Loughry WJ, Prodöhl PA, McDonough CM, Avise JC (1998). Polyembryony in armadillos. Am Sci.

[4] Ivanova-Kasas OM, Counce SJ, Waddington CH (1972). Polyembryony in insects. Developmental systems.

[5] Uematsu H (1978). The effective temperature and seasonal prevalence of *Thysanoplusia intermixta* (WARREN) (Lepidoptera: Noctuidae) Proc. Assoc PI Prot Kyushu.

[6] Baehrecke EH, Strand MR (1990). Embryonic morphology and growth of the polyembryonic parasitoid *Copidosoma floridanum* (Ashmead) (Hymenoptera : Encyrtidae) international J. Insect Morphol Embryol.

[7] Nakaguchi A, Hiraoka T, Endo Y, Iwabuchi K (2006). Compatible invasion of a phylogenetically distant host embryo by a hymenopteran parasitoid embryo. Cell Tissue Res.

[8] Strand MR (1989). Oviposition behavior and progeny allocation of the polyembryonic wasp *Copidosoma floridanum* (Hymenoptera: Encyrtidae). J Insect Behav.

[9] Grbic M, Nagy LM, Carroll SB, Strand M (1996). Polyembryonic development: insect pattern formation in a cellularized environment. Development.

[10] Iwabuchi K (1995). Effect of juvenile hormone on the embryogenesis of a polyembryonic wasp, *Copidosoma floridanum*, in vitro. Vitr Cell Dev Biol - Anim J Soc Vitr Biol.

[11] Gilbert LI, Granger NA, Roe RM (2000). The juvenile hormones: historical facts and speculations on future research directions. Insect Biochem Mol Biol.

[12] Truman JW, Riddiford LM (1999). The origins of insect metamorphosis. Nature.

[13] Adam G (2003). The retinoic-like juvenile hormone controls the looping of left-right asymmetric organs in *Drosophila*. Development.

[14] Germain P, Germain P, Chambon P, Eichele G, Evans RM, Lazar MA (2006). International Union of Pharmacology. LXIII Retinoid X Receptors Pharmacological Reviews.

[15] Harmon MA, Boehm MF, Heyman RA, Mangelsdorf DJ (1995). Activation of mammalian retinoid X receptors by the insect growth regulator methoprene. Proc Natl Acad Sci.

[16] Jones G, Jones D (2000). Considerations on the structural evidence of a ligand-binding function of ultraspiracle, an insect homolog of vertebrate RXR. Insect Biochem Mol Biol.

[17] Grenier E, Levy E, Tremblay E, Delvin E, Garofalo C, Sane A (2007). Effect of retinoic acid on cell proliferation and differentiation as well as on lipid synthesis, lipoprotein secretion, and apolipoprotein biogenesis. Am J Physiol Liver Physiol.

[18] Tabunoki H, Bono H, Ito K, Yokoyama T (2016). Can the silkworm (*Bombyx mori*) be used as a human disease model?. Drug Discov Ther.

[19] Chen X, Tomchick DR, Kovrigin E, Araç D, Machius M, Sü TC (2002). Three-dimensional structure of the Complexin/SNARE complex energy of SNARE complex formation may be used to overcome the energy barrier required to bring the synaptic vesicle and plasma membranes together, perhaps. Neuron.

[20] Mohrmann R, De Wit H, Verhage M, Neher E, Sørensen JB (2010). Fast vesicle fusion in living cells requires at least three SNARE complexes. Science.

[21] Südhof TC (2013). Neurotransmitter release: the last millisecond in the life of a synaptic vesicle. Neuron.

[22] Grbić M, Nagy LM, Strand MR (1998). Development of polyembryonic insects: A major departure from typical insect embryogenesis. Dev Genes Evol.

[23] Nakamura F, Stossel TP, Hartwig JH (2011). The filamins: organizers of cell structure and function. Cell Adhes Migr.

[24] Niwa R, Nagata-Ohashi K, Takeichi M, Mizuno K, Uemura T (2002). Control of actin reorganization by slingshot, a family of phosphatases that dephosphorylate ADF/cofilin. Cell..

[25] Yang JT, Rayburn H, Hynes RO (1995). Cell adhesion events mediated by alpha 4 integrins are essential in placental and cardiac development. Development.

[26] Hayashi Y (1961). Studies on the xanthine oxidase system in the silkworm, *Bombyx mori* L. (III) changes of the uric acid content and xanthine dehydrogenase activity in the silkworm egg during embryonic development. Bull. Sericul. Exp. Stat..

[27] Chen GL, Ye T, Chen HL, Zhao ZY, Tang WQ, Wang LS (2017). Xanthine dehydrogenase downregulation promotes TGFβ signaling and cancer stem cell-related gene expression in hepatocellular carcinoma. Oncogenesis.

[28] Taibi G, Di Gaudio F, Nicotra CMA (2008). Xanthine dehydrogenase processes retinol to retinoic acid in human mammary epithelial cells. J Enzyme Inhib Med Chem.

[29] Zhou X (2008). Riddiford LM: rosy function is required for juvenile hormone effects in *Drosophila melanogaster*. Genetics.

[30] Kawasaki K, Ikeuchi M, Hidaka T (1987). Laboratory rearing method for *Acanthoplusia agnata* (Lepidoptera: Noctuidae) without change of artifical diet. Japanese J Appl Entomol Zool.

[31] Iwabuchi K (1991). Early Embronic development of a Polyembryonic wasp, *Litomastix maculata* ISHII, in vivo and in vitro. Appl Entomol Zool.

[32] Pertea M, Kim D, Pertea GM, Leek JT, Salzberg SL (2016). Transcript-level expression analysis of RNA-seq experiments with HISAT, StringTie and Ballgown. Nat Protoc.

[33] Wysoker A, Fennell T, Marth G, Abecasis G, Ruan J, Li H (2009). The sequence alignment/map format and SAMtools. Bioinformatics.

[34] Liao Y, Smyth GK, Shi W (2013). The subread aligner: fast, accurate and scalable read mapping by seed-and-vote. Nucleic Acids Res.

[35] Liao Y, Smyth GK, Shi W (2014). featureCounts: an efficient general purpose program for assigning sequence reads to genomic features. Bioinformatics.

[36] Sun J, Nishiyama T, Shimizu K, Kadota K (2013). TCC: an R package for comparing tag count data with robust normalization strategies. BMC Bioinformatics.

