# Supplementary figures and images for: Analysis of molecular mechanism for acceleration of polyembryony using gene functional annotation pipeline in Copidosoma floridanum
Source: BMC Genomics. 2020 Feb 11;21:152. doi: 10.1186/s12864-020-6559-3 (PMC7014612; doi:10.1186/s12864-020-6559-3)

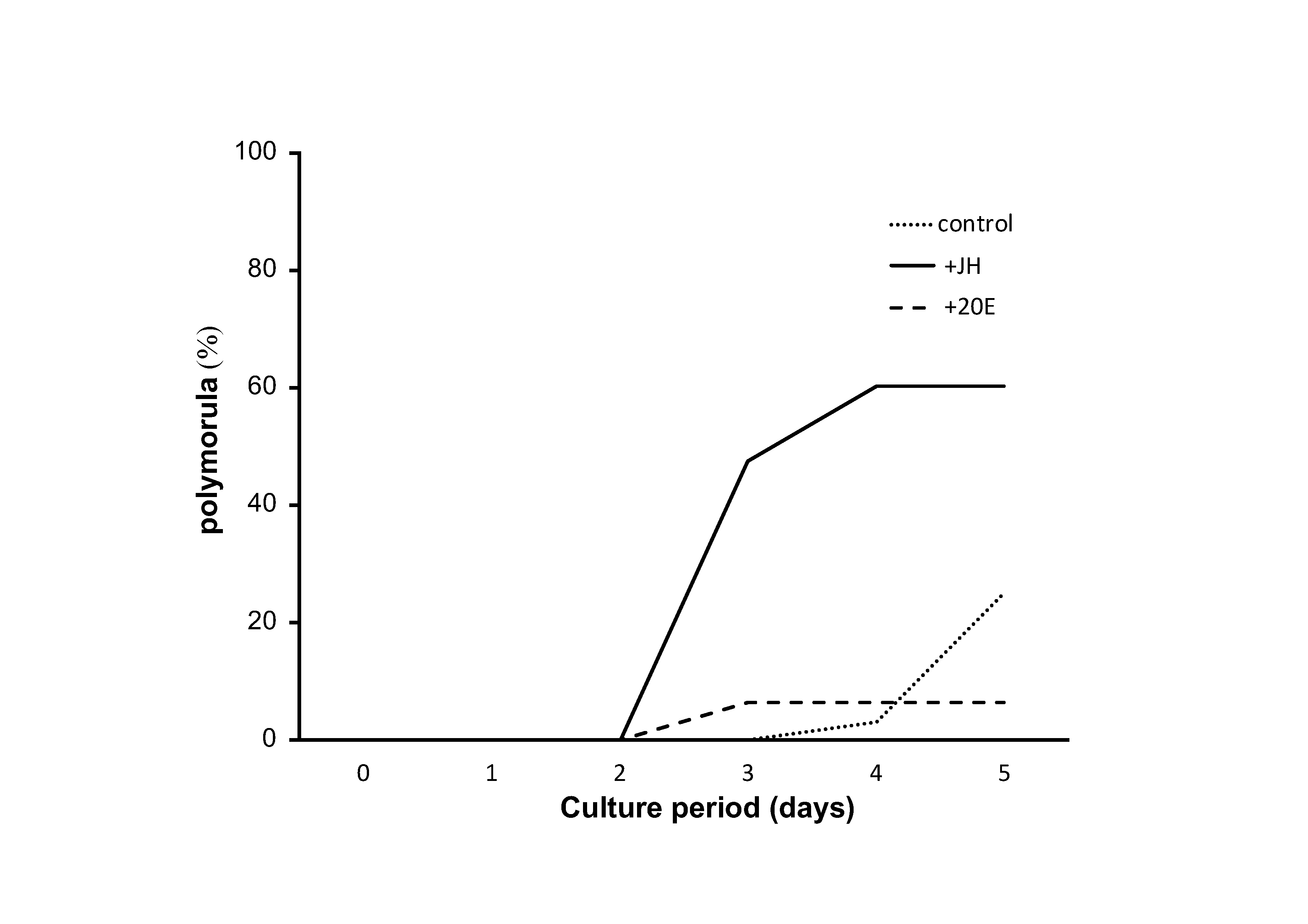

Supplement: Supplementary file 2 — Additional file 2: Figure S1. Polymorula was accelerated by the juvenile hormone (JH) treatment in the culture condition. The early embryo of the two-cell stage was cultured with or without JH, or Ecdysone. The number of polymorula was counted and plotted on the graph. Solid line, JH treatment; dashed line, treatment without JH; wide dashed line, treatment Ecdysone, vertical axis, a rate of polymorula (%); horizontal axis, the culture period (day). [file 12864_2020_6559_MOESM2_ESM.tiff]

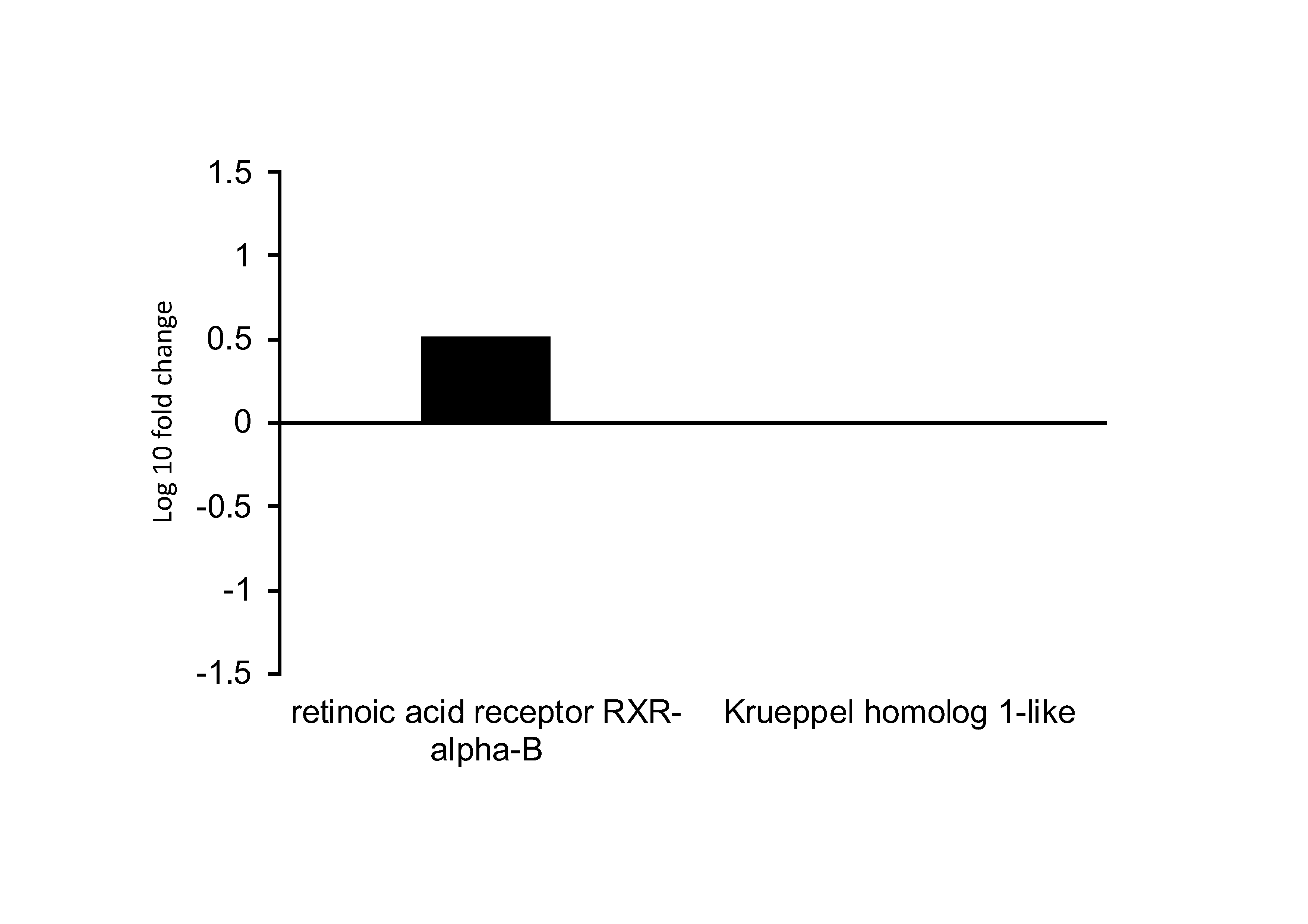

Supplement: Supplementary file 3 — Additional file 3: Figure S2. Expression of Krueppel homolog 1-like genes, and retinoic acid receptor RXR-alpha-B in juvenile hormone (JH) treatment of molura. The y-axis indicates the ratio of the average Transcripts Per Kilobase Million (TPM) values for molura between the control and JH treatment groups. [file 12864_2020_6559_MOESM3_ESM.tiff]
